# Lower Serum Zinc Levels in Patients with Multiple Sclerosis Compared to Healthy Controls

**DOI:** 10.3390/nu10080967

**Published:** 2018-07-26

**Authors:** Marc Pawlitzki, Julia Uebelhör, Catherine M. Sweeney-Reed, Heike Stephanik, Juliane Hoffmann, Anke Lux, Dirk Reinhold

**Affiliations:** 1 Department of Neurology, Otto-von-Guericke-University, 39120 Magdeburg, Germany; julia.uebelhoer93@gmail.com (J.U.); catherine.sweeney-reed@med.ovgu.de (C.M.S.-R.); heike.stephanik@med.ovgu.de (H.S.); 2 Institute of Clinical Chemistry and Pathobiochemistry, Otto-von-Guericke-University, 39120 Magdeburg, Germany; juliane.hoffmann@med.ovgu.de; 3 Department for Biometrics and Medical Informatics, Otto-von-Guericke-University, 39120 Magdeburg, Germany; anke.lux@med.ovgu.de; 4 Institute of Molecular and Clinical Immunology, Otto-von-Guericke-University, 39120 Magdeburg, Germany; dirk.reinhold@med.ovgu.de

**Keywords:** multiple sclerosis, zinc, disease-modifying drugs

## Abstract

Objective: Diminished blood levels of zinc have been reported to be associated with T-cell-mediated autoimmunity, which has been implicated in multiple sclerosis (MS). We aimed to compare the distribution of serum zinc status in MS patients with that in healthy controls (HCs) and to investigate a potential correlation with clinical state, through analysis of serum zinc concentration in MS patients suffering from different disease subtypes. Methods: Serum zinc concentrations of 133 patients with relapsing (RMS) and 18 patients with the progressive form of MS (PMS), according to the McDonald criteria of 2010, were measured. Clinical status was quantified using the Expanded Disability Status Scale (EDSS). Zinc concentrations were also determined in the sera of 50 HCs, matched for age and sex at a group level. Results: MS patients showed significantly lower zinc concentrations (mean (SD)) than HCs (12.5 (2.1) µmol/L vs. 14.6 (2.3) µmol/L, *p* < 0.001). In contrast, we did not find any difference between RMS (12.4 (2.0) µmol/L) and PMS (13.0 (3.0) µmol/L) cases (*p* = 0.8). Patients receiving disease-modifying treatment showed lower mean (SD) serum zinc levels than untreated cases (12.3 (1.9) µmol/L vs. 13.5 (3.2) µmol/L, *p* < 0.03). Zinc levels were not related to disease duration, EDSS, annual relapse rate, or the median number of relapses. Conclusions: The data suggest that a diagnosis of MS is related to lower serum zinc concentrations than in HCs, and concentrations were lower still under disease-modifying therapy. However, zinc levels did not predict disease subtypes or disability status.

## 1. Introduction

Multiple sclerosis (MS) is a neurological, T cell-mediated autoimmune disease characterized by an imbalance of proinflammatory and anti-inflammatory factors to the detriment of the latter [1]. The identification of potential nutritional supplements as treatment options is of growing interest, particularly for patients in whom disease-modifying drugs do not hinder disease progression or when nutritional deficiencies prevail [2].

Zinc is an essential trace element, which is required by a large number of enzymes, proteins, and transcription factors [3,4,5]. Indeed, abnormal lower blood levels of zinc are related to increased T-cell counts as well as T-cell mediated autoimmunity [3]. Conversely, it remains a matter of debate to what extent zinc deficiency could play a critical role in MS, especially as existing studies in MS patients reveal a large span of blood zinc concentrations, ranging from slightly lower to higher levels in contrast to healthy controls (HCs) [6]. The observed range in zinc levels might be related to the majority of these MS studies comprising small cohorts or including patients at different disease stages, limiting the extent to which the conclusions drawn regarding serum zinc levels in MS can be extended to MS patients in general. 

Here, we conducted a cross-sectional study, measuring zinc blood concentrations in a large cohort of patients suffering from different subtypes of MS to uncover how the serum zinc status is distributed in MS patients compared to HCs and how it may be related to the current clinical state.

## 2. Material and Methods

### 2.1. Patients, Controls, and Clinical Assessment

Serum zinc concentration was determined for 133 patients with relapsing (RMS) (*n* = 9 clinically isolated syndrome, *n* = 124 relapsing remitting MS) and 18 patients with the progressive form of MS (PMS) (*n* = 10 secondary progressive MS, *n* = 8 primary progressive MS) according to the McDonald criteria (2010) [7]. The patients were consecutively recruited through the Department of Neurology at the Otto-von-Guericke-University Magdeburg. Disease duration was defined as the time in years between diagnosis and blood sampling. Clinical scoring was available from the clinical record for all patients using the Expanded Disability Status Scale (EDSS) [8]. Previous patient reports were also examined, and clinical examination was performed at the time of blood sampling. These evaluations were used in an exploratory analysis, to search for symptoms or signs that could be potentially associated both with lower zinc levels and MS disease, such as depression [9,10], diuretic use (angiotensin-converting-enzyme inhibitors, angiotensin 2 receptor antagonists or thiazide diuretics) [11], diabetes [12], or vegetarian diet [13].

Additionally, zinc concentrations were measured in the sera of *n* = 50 age- and sex-matched controls without a history of neurological or psychiatric disorders or diabetes (HCs), who were recruited from medical staff and their families and the community.

To exclude potential confounding factors, all participants (i) were evaluated in the absence of signs of clinical infection or an acute inflammatory relapse (patients only), (ii) should not have received any type of corticosteroids in the preceding 4 weeks, (iii) should not be pregnant, and (iv) were asked whether they were taking zinc supplements.

The study was approved by the local ethics committee of the Otto-von-Guericke-University Magdeburg, Germany (No 80/16), and all participants provided written informed consent.

### 2.2. Ethical Publication Statement

We confirm that we have read the journal’s position on issues involved in ethical publication and affirm that this report is consistent with those guidelines.

### 2.3. Zinc Measurement

Venous blood samples (6 mL) were collected in specially obtained metal-free tubes (BD Vacutainer, Ref. 368380, BD Vacutainer®, Franklin Lakes, NJ, USA) from all participants during the morning (between 08:00 and noon) to avoid a potential confounding effect of circadian fluctuation [14]. The blood samples were immediately transferred to the Institute of Clinical Chemistry and Pathobiochemistry Magdeburg, for separation by centrifugation. The serum zinc level was quantified using an atomic absorption iCE3500 spectrophotometer (ThermoFisher Scientific, Waltham, MA, USA). 

### 2.4. Statistical Analysis

**Figure 1:**
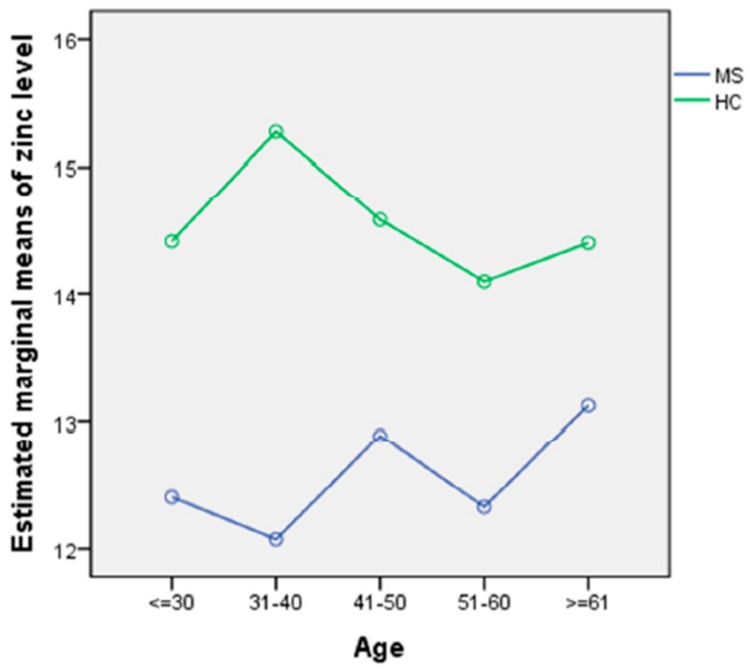
HCs = healthy controls, MS = multiple sclerosis. The profile plots show the estimated marginal means of zinc values separated for the two groups. There was no interaction between age and group. There was a main effect of group and no effect of age.

Statistical analysis was conducted using SPSS 21 (ISPSS Inc, Chicago, IL, USA). The groups (MS, HC) were compared with respect to categorical variables using a *χ*^2^-test. Independent sample *t*-tests were performed for continuous variables to determine group differences between patients and controls, and Mann-Whitney-U tests were applied when the variables were not normally distributed. A two-way ANOVA applying pairwise Dunn-Bonferroni post-hoc testing was conducted with age and zinc level as the dependent variables and group (HC vs. RMS vs. PMS) as the independent variable. Moreover, a univariate analysis of variance, including a diagram of the estimated marginal means (see Figure 1) was performed to evaluate between-subject-effects and the effect of age on zinc. Spearman correlations (Spearman’s rank correlation coefficient = rho) were calculated between zinc levels, age, sex, and clinical values, including EDSS, relapse rate, and disease duration. *p*-values ≤ 0.05 were deemed to be statistically significant. 

## 3. Results

### 3.1. Cohorts

Demographic and clinical data from the cohort are provided in Table 1. Thirteen MS patients (*n* = 12 RMS, *n* = 1 PMS) and one HC reported taking regular supplementary zinc preparations. Three MS patients used diuretics. Moreover, the rates of diabetes and vegetarianism were less than 5% in both groups. Mean (SD) age and sex did not differ between HCs (43 [14] years, 76% female) and MS patients (43 [12], 75%). As expected, mean (SD) age and sex distributions of PMS patients (55 [9], 100%) differed (*p* < 0.001, *p* = 0.03) from HCs (*p* = 0.001, *p* = 0.03) and RMS cases (42 [11], 71%). Mean (SD) disease duration was 10 years [8] in the MS patients and did not differ between the subgroups (RMS = 9 [8], PMS 13 [11], *p* = 0.1). Median EDSS at the time of blood sampling was lower in RMS patients (2.5) than in PMS patients [6] (*p* < 0.001).

Considering the whole patient group, *n* = 131 (87%) received disease-modifying treatment (DMT). Patients with RMS (*n* = 118; 89%) received the following DMTs: interferon beta (*n* = 18), glatiramer acetate (*n* = 24), teriflunomide (*n* = 5), dimethylfumarate (*n* = 12), fingolimod (*n* = 19), natalizumab (*n* = 34), alemtuzumab (*n* = 3), rituximab (*n* = 2), and daclizumab (*n* = 1). Of the patients diagnosed with PMS, *n* = 13 (72%) were treated with DMTs, including interferon beta (*n* = 5), glatiramer acetate (*n* = 6), fingolimod (*n* = 1) and daclizumab (*n* = 1). The median relapse rate, annualized relapse rate (ARR), and EDSS did not differ between treated and untreated cases.

### 3.2. Serum Zinc Levels

Univariate analysis underlined the group difference in particular (*p* < 0.001), and the absence of an effect of age on zinc levels (*p* = 0.8). There was no interaction between age and group (*p* = 0.5). The profile plot did not show any deviation of values depending on age (Figure 1). 

**Figure 2:**
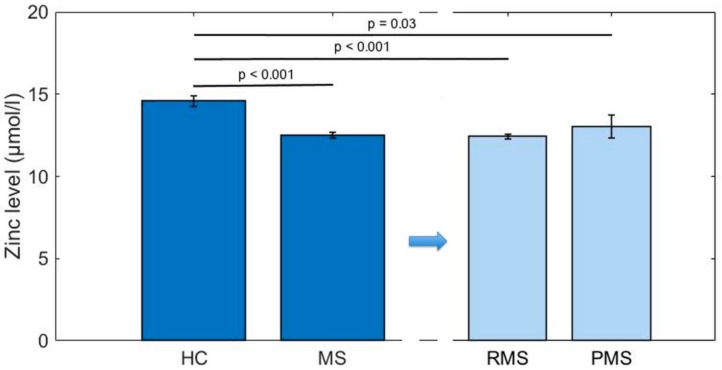
Mean serum zinc levels with standard error of the mean. HC = healthy controls, MS = multiple sclerosis, PMS = progressive multiple sclerosis, RMS = relapsing multiple sclerosis. Group comparisons were conducted using ANOVA with post-hoc Dunn-Bonferroni-testing. Significance threshold: *p* < 0.05. PMS and RMS patients showed lower zinc blood levels than HCs, while zinc levels between PMS and RMS patients did not differ.

MS patients had significantly lower mean (SD) zinc concentrations than HCs (12.5 (2.1) µmol/L vs. 14.6 (2.3) µmol/L, *p* < 0.001). No difference was observed between RMS (12.4 (2.0)) and PMS (13.0 (3.0)) cases (*p* = 0.8). Patients with RMS and PMS also showed significantly lower mean zinc values than HCs when analyzed separately (*p* < 0.001, *p* = 0.03 respectively) (Table 1, Figure 2). Treated patients showed lower mean (SD) zinc levels than untreated cases (12.3 (1.9) µmol/L vs. 13.5 (3.2) µmol/L, *p* = 0.03). Zinc levels were not related to disease duration (*rho* = 0.02, *p* = 0.8), EDSS (*rho* = 0.09, *p* = 0.3), annual relapse rate (*rho* = −0.1, *p* = 0.2), the median number of relapses (*rho* = −0.1, *p* = 0.2), or depression incidence (*rho* = −0.02, *p* = 0.9) (Table 1). The findings remained unchanged when participants with zinc supplementation, diabetes, diuretic use, or vegetarian diet were excluded, or when dividing the cohort into male and female subgroups. Moreover, zinc-supplemented MS patients did not show lower EDSS scores or higher rates of depression.

Mean (SD) zinc levels did not differ between females and males (12.9 (2.5) µmol/L vs. 13.5 (1.8) µmol/L, *p* = 0.09).

Abnormal zinc levels were identified in only five patients (three with lower, two with higher) and four HCs (increased) according to the local laboratory reference range (9.0–18.0 µmol/L). 

## 4. Discussion

In the last decades, serum zinc levels have been examined in several small study cohorts to investigate a potential role for zinc in MS, with the suggestion that lower zinc blood levels are associated with higher levels of disease-related disability [6].

In the present study, we investigated serum zinc levels in the largest cohort of MS patients to our knowledge so far. The patient group was characterized by a relapsing or progressive disease course and was compared with HCs. MS diagnosis was related to lower serum zinc concentrations compared to HCs, but without marked zinc deficiency. This group difference could not be further explained by other variables, including age and sex. However, zinc levels did not differ according to disease subtype or relate to clinical measures. Interestingly, treated MS patients had lower zinc values than untreated cases.

Our findings of significantly lower serum zinc values in MS patients compared to HCs are in line with some previous findings [15,16], but several investigations have reported either no difference [17,18] or higher zinc values [19] in MS patients. The heterogeneity in the literature to date, however, is likely due to small cohort sizes [18,20] or the absence of a representative control group [21]. We aimed to overcome these shortcomings by recruiting a large patient cohort with matched controls, characterized by a wide range of ages. Interestingly, we did not detect a relationship between age and zinc values. Several studies in which no difference was seen in zinc levels between MS patients and controls had a cohort comprising a high female:male ratio, which was considered to be in line with findings of significantly lower zinc levels in male than female MS patients and thus potentially masked a difference between groups [22]. In contrast, we showed lower zinc levels compared to HCs both in our RMS and PMS cohorts, the latter of which included only female patients. 

Lower zinc values in the MS patients could be due to increased upregulation of zinc-dependent matrix metalloproteinases, which have shown higher levels during the active disease stage of MS patients [23], and such an upregulation might explain the reported lower zinc levels in RMS cases compared to MS patients with chronic (and non-active) disease [24]. Zinc deficiency is reported to induce an imbalance between Th1 and Th2 functions [25] and hinder down-regulation of Th17 lymphocytes [26], which have been suggested to be major mechanisms contributing to the pathogenesis of MS autoimmunity [27] and are highly elevated within MS blood samples [28].

Interestingly, treated MS patients had significantly lower zinc values than untreated patients, which could be explained by the high rate of liver metabolism of the most commonly used DMTs in MS [29], leading to an impairment of zinc homeostasis [30]. On the other hand, patients receiving treatment may systematically differ from those not on medication. Although their clinical states did not differ at the time of the study, it is plausible that they had more severe symptoms and or signs of disease at the time of commencing medication.

In line with recent studies, we did not find any relationship between the patients’ clinical measures (e.g., EDSS, disease duration, depression) and zinc concentration. Zinc level alterations have been postulated to reflect cumulative underlying immunological processes that cannot be mirrored by a disability score taken at a single time point. In particular, the absence of an acute clinical deterioration could explain the generally lower, though still within the normal range, zinc levels, which can be observed in acute clinical relapses or due to acute prednisolone therapy [23,31]. Despite the absence of a relationship between zinc levels and depression prevalence, recent preliminary results suggest that zinc supplementation in MS can improve psychiatric symptoms, which could be due to a zinc deficiency in both disease entities [32].

Although we found a significant difference between the two groups, our population did not show a significant zinc deficiency. We consider here the possible reasons for the lack of zinc deficiency as well as differences between the zinc ranges observed in the current study and those reported elsewhere. The normal zinc range might be explained by the low rate of zinc-reducing confounding factors such as diabetes [12] or vegetarian diet [13], as well as the exclusion of pregnant patients [33] and those receiving prednisone treatment [31]. Had these factors been present in our cohort and equally distributed across the MS patients and HCs, we might expect that the inter-group difference we detected would still have been present, but that the zinc levels in the MS patient group would have fallen below the normal range. The EDSS was not considered in previous studies, but the median EDSS of 3 in our cohort suggests an unrestricted ability to walk and thus, a participation in daily life. Higher degree of disability, especially limited mobility, can reduce daily intake of nutrients resulting in zinc deficiency [34]. The patients and HCs were recruited from the same region of Germany, where socioeconomic status has a minimal effect on nutritional status. A larger variation in zinc status is expected between low- and high-income countries and could explain the difference in the zinc level range reported here compared with studies conducted elsewhere [35,36]. As additional confounders, the interaction with highly elevated blood copper levels [37], malnutrition, and heavy metal pollution [38] in developed countries could explain differences between the study outcomes as well. Normal, but lower values in the absence of bias in our MS patient cohort might thus be explained by an autoimmune disease-related feedback loop between inflammation, zinc uptake, and zinc reduction [39].

In addition, zinc-substituting patients did not show higher mean zinc values (*p* = 0.5). This finding is consistent with inadequate zinc supplementation but could also be cautiously interpreted as suggesting an active process in MS patients by which zinc levels are reduced, rather than low zinc playing a causal role in or having an indirect association with MS [3,6].

The strengths of our study comprise the inclusion of MS patients at different disease stages, the absence of potential clinical confounders, e.g. acute relapse or prednisolone treatment, and the use of metal-free blood-sampling tubes. Limitations include a relatively small sample size of PMS patients, which did not allow the splitting of the cohort into secondary and primary progressive case groups, and the ongoing immunological drug therapy, which was not interrupted for ethical reasons. 

## 5. Conclusions

In summary, we identified an association between MS diagnosis and lower zinc levels. This finding is consistent with the lower relapse rate and faster remission of relapse-induced disability observed in mice with experimental autoimmune encephalomyelitis, the most commonly used experimental model for MS, when given zinc supplements [40]. Future studies should focus on prospective, double-blinded clinical trials to investigate personalized zinc supplementation for MS patients. 

## Figures and Tables

**Table 1:** *n* = number of participants; unless otherwise reported mean [standard deviation] is given. ARR = annualized relapse rate, HC = healthy controls, MS = multiple sclerosis, PMS = progressive multiple sclerosis, RMS = Relapsing multiple sclerosis. Disease duration was defined as the timespan between diagnosis and the date of blood sample. For group comparisons a *c*^2^-test, an independent-samples t-test or a Mann-Whitney-U test were performed. Moreover, ANOVA with post-hoc Bonferroni-testing was conducted. *p*-values < 0.05 were deemed to be statistically significant.

Title	HC (*n* = 50)	MS (*n* = 151)	RMS (*n* = 133)	PMS (*n* = 18)	*p*-Values			
					HC vs. MS	HC vs. RMS	HC vs. PMS	RMS vs. PMS
Age at zinc measure (years)	43 [14] (19–82)	43 [12] (18–80)	42 [11] (18–76)	55 [9] (40–80)	1.0	0.5	0.001	<0.001
Female sex, *n* (%)	38 (76)	113 (75)	95 (71)	18 (100)	0.9	0.03	0.03	0.03
Disease duration (years)	-	10 [8] (0–35)	9 [8] (0–29)	13 [11] (0–35)	-	-	-	0.1
Vegetarian, *n* (%)	2 (4)	8 (5)	7 (5)	1 (5)	0.8	0.9	0.9	0.9
Diabetes, *n* (%)	0 (0)	7 (5)	6 (5)	1 (5)	0.1	0.9	0.9	0.9
Hematocrit	-	0.42 (0.03)	0.42 (0.03)	0.42 (0.02)	-	-	-	0.7
Median EDSS	-	3 (0–8)	2.5 (0–7.5)	6.0 (2–8)	-	-	-	<0.001
Median relapse, *n*	-	2 (0–20)	2 (0–20)	1 (0–6)	-	-	-	0.003
ARR	-	0.5 (0.5)	0.5 (0.5)	0.2 (0.2)	-	-	-	0.001
Treatment, *n* (%)	-	131 (87)	118 (89)	13 (72)	-	-	-	0.05
Depression, *n* (%)	-	39 (26)	34 (26)	5 (28)	-	-	-	0.5
Zinc level (µmol/L)	14.6 (2.1) (9.5–19.3)	12.5 (2.1) (8.7–24.8)	12.4 (2.0) (8.7–24.8)	13.0 (3.0) (8.8–20.3)	<0.001	<0.001	0.03	0.8
